# Supplementary material for: Identification of photoperiod-induced specific miRNAs in the adrenal glands of Sunite sheep (Ovis aries)
Source: Front Vet Sci. 2022 Jul 22;9:888207. doi: 10.3389/fvets.2022.888207 (PMC9354845; doi:10.3389/fvets.2022.888207)
Supplement: Supplementary file 1 [file Data_Sheet_1.ZIP › Supplementary file/miRDeep2 file.zip!/miRDeep2 file/Novel_Structure/SPLP42A3/Novel_331.pdf]

Provisional ID	: NC_019484.2_37877
Score total	: 2.7
Score for star read(s)	: -1.3
Score for read counts	: 0
Score for mfe	: 2.4
Score for randfold	: 1.6
Score for cons. seed	:
Total read count	: 817
Mature read count	: 817
Loop read count	: 0
Star read count	: 0

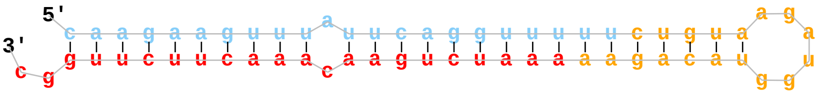

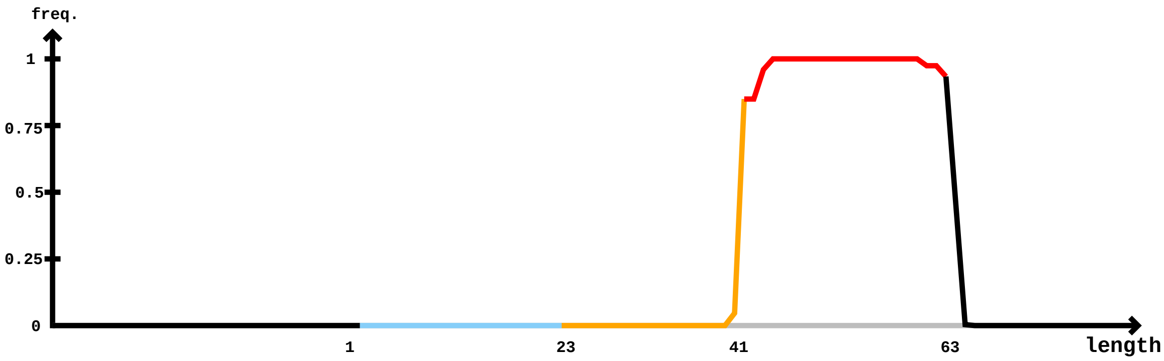

Star Mature

```
5'- uucaucuguaaauccaauauacuggguuggccaagaaguuuauucagguuuuucuguaagaugguacagaaaaaucugaacaaacuucuuggccaacccaauacuuaauucu   -3'    exp
    ......................(((((((((((((((((((.((((((((((((((((......)))))))))))))))).))))))))))))))))))............   reads   mm   sample
    ......................................................................aaaaucugaacaaacuuUuu......................   18      1    seq
    ......................................................................aaaaucugaacaaacuuUuugg....................   19      1    seq
    ......................................................................aaaaucugaacaaacuucuuggc...................   1       0    seq
    .......................................................................aaaucugaacaaacuuUuu......................   3       1    seq
    .......................................................................aaaucugaacaaacuuUuugg....................   14      1    seq
    .......................................................................aaaucugaacaaacuuUuuggc...................   637     1    seq
    .......................................................................aaaucugaacaaacuucuuggc...................   2       0    seq
    .........................................................................aucugaacaaacuuUuuggc...................   16      1    seq
    .........................................................................aucugaacaaacuucuuggc...................   1       0    seq
    .........................................................................aucugaacaaacuuUuuggcc..................   73      1    seq
    ..........................................................................ucugaacaaacuuUuuggcc..................   30      1    seq
    ..........................................................................ucugaacaaacuuUuuggcca.................   3       1    seq
```
